# Comparison of Protein Gut Samples from *Rhipicephalus* spp. Using a Crude and an Innovative Preparation Method for Proteome Analysis

**DOI:** 10.3390/vetsci5010030

**Published:** 2018-03-14

**Authors:** Thomas P. Karbanowicz, Amanda Nouwens, Ala E. Tabor, Manuel Rodriguez-Valle

**Affiliations:** 1 Queensland Alliance for Agriculture & Food Innovation, University of Queensland, St. Lucia 4072, Australia; t.karbanowicz@uq.edu.au (T.P.K.); a.lewtabor@uq.edu.au (A.E.T.); 2 School of Chemistry and Molecular Biosciences, University of Queensland, St. Lucia 4072, Australia; a.nouwens@uq.edu.au; 3 Centre for Comparative Genomics, Murdoch University, Perth 6150, Australia

**Keywords:** cattle tick, proteomics, tick vaccine candidates, gut surface antigens

## Abstract

Tick populations are controlled through the application of chemical pesticides. However, the rise in chemical resistance has prompted the investigation of other control methods such as the use of tick vaccines. Proteomic analysis provides valuable information about the possible function and localization of proteins, as candidate vaccine proteins are often either secreted or localized on the cell-surface membrane. Progress in the utilization of proteomics for the identification of novel treatment targets has been significant. However, their use in tick-specific investigations is still quite novel, with the continual development of tick-specific methodologies essential. In this study, an innovative sample preparation method was utilized to isolate epithelial cells from tick midguts to identify the membrane-bound proteins. Proteomic analysis was conducted comparing crude and innovative sample preparation methods with 692 and 1242 tick-specific proteins, 108 and 314 surface proteins respectively, isolated from the midguts of semi-engorged *Rhipicephalus microplus* adult female ticks. This research reports a novel preparation protocol for the analysis of tick midgut proteins which reduces host protein contamination.

## 1. Introduction

Ticks are obligate hematophagous ectoparasites requiring a blood meal prior to molting to the next stage of development, including egg laying. Such ticks have evolved mechanisms to allow for uninterrupted blood feeding, making them the ideal vector for many pathogenic diseases. *Rhipicephalus microplus*, the cattle tick, is the most significant ectoparasite in tropical and sub-tropical regions with associated economic losses estimated at US $22–30 billion annually [1]. *Rhipicephalus microplus* is a one-host tick species with all life stages feeding on the same bovine host, resulting in a two-fold effect of parasitism. The direct mechanism of tick feeding leads to blood loss, lesions, overall reduction in both weight and milk production; and the tick acting as a vector transmitting pathogens such as bovine tick fever (babesiosis and anaplasmosis), and equine piroplasmosis [2,3,4,5]. Tick populations are controlled through the application of chemical pesticides, however concerns over their continued use have escalated. Increases in chemically resistant tick populations, environmental awareness, and food and animal product contamination have led to an interest in the development and use of alternative control methods [6,7].

The identification of potential candidate vaccine antigens remains an important goal for researchers with the application of “omics” technologies, particularly proteomics and vaccinomics, contributing to the identification of several potential antigens [8,9]. Typically, the best candidates for recombinant vaccine are membrane-bound or secreted proteins as they are the first molecules to encounter host immune responses [10,11,12]. Despite tremendous efforts in tick vaccine research, there have only been two commercial tick vaccines, TickGard*PLUS*™ and Gavac™, developed in the early 1990s. These vaccines were based on recombinant *R. microplus* Bm86/Bm95 antigens, Glycosylphosphatidylinositol (GPI)-linked glycoproteins, located on the midgut of *R. microplus* [13,14]. The vaccines showed effective control of *R. microplus* and *Rhipicephalus annulatus* by reducing the number, weight, and reproductive capacity of engorging females, resulting in a reduced larval infestation in subsequent tick generations [15]. Bm86-based vaccines were poorly adopted by cattle industries as they require 3–4 boosts per year, are not effective against all tick stages, and have poor or no efficacies in geographical regions such as Brazil, Argentina, and South Africa [16,17,18]. Consequently, the distribution of the Australian vaccine, TickGard*PLUS*™, concluded in 2010. 

Progress in the utilization of proteomics for identification of novel vaccine targets has been significant, as improvements in proteomic methodology, sample preparation, peptide/protein separation methods, mass spectrometry data collection, and data analysis and interpretation have vastly improved [19,20,21]. Despite these advances, the application of proteomics in tick investigations is novel with 60% of tick proteomic studies published in the last 5 years [22]. Tick-specific proteomics have been primarily focused upon the mid-gut of economically-significant tick species such as *R. microplus*, *Ornithodoros moubata* and *Ornithodoros erraticus*, with increasing interest in the salivary glands and salivary gland extracts of other tick species [23,24,25]. Current limitations behind the application of proteomics for tick research include the scarce amount of fresh biological samples (i.e., particular tick organs) with representative biological replicates [26], lack of comprehensive tick species-specific sequence databases, and the coexistence of proteins from ticks, hosts and vector-borne symbionts or pathogens [22]. 

These limitations require the continual development of tick-specific methodologies to effectively apply proteomic technologies. This study aims to utilize the membrane-bound protein isolation methods reported by Karbanowicz et al. 2017 [27], in comparison to traditional crude extraction methods [28] to investigate the membrane proteins within the midguts of semi-engorged *R. microplus* at the exclusion of host proteins.

## 2. Materials and Methods 

### 2.1. Single Epithelial Cell Dissociation from the Tick Gut Epithelium of R. microplus

**Figure 1:**
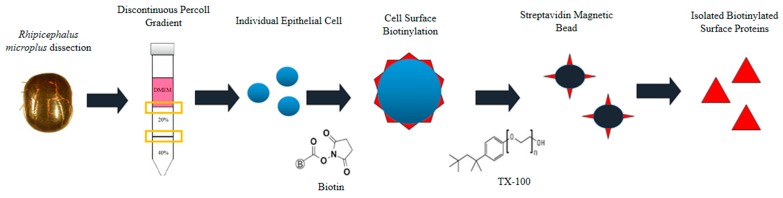
Schematic representation of the isolation of biotinylated proteins present on the surface of *Rhipicephalus microplus* midgut cells. Figure source [27].

Single epithelial cells were dissociated from crude tick gut of *R. microplus* described by Karbanowicz et al. 2017 [27] (Figure 1), with the following amendments. One hundred semi-engorged *R. microplus* adult female ticks were collected from infested Hereford cattle (Biosecurity Tick Colony of the Queensland Department of Agriculture and Fisheries at the Queensland Animal Science Precinct, UQ Gatton Campus, Queensland, Australia) and immediately prepared for dissection by sticking the tick ventral side down onto a petri dish using a drop of super glue (UHU, Wetherill Park, NSW, Australia). Ticks were left to dry for ~1–2 min, prior to submerging in 1× phosphate buffered saline (PBS). Under a dissection microscope (Olympus SZX7, Edmund Optics, Singapore, Singapore), a size 11 scalpel was used to cut from the top of the eyes to the bottom festoons on both sides of the tick. Tick internal organs were exposed by using sterile forceps to remove the scutum and alloscutum, with the trachea removed and disposed. Tick guts were dissected from the carcass and stored in ice-cold Hank’s Balanced Salt Solution (HBSS) without calcium chloride and magnesium sulfate (ThermoFisher Scientific, Scoresby, Australia). Protease-inhibitor cocktail (PIC) (Sigma-Aldrich, Castle Hill, NSW, Australia) was included to prevent enzymatic activity. 

Dissected guts were placed in a 70-µm cell strainer (BD Falcon, North Ryde, Australia) and washed with 50 mL ice cold HBSS with PIC until the solution ran clear. Guts were re-suspended in 30 mL ice cold HBSS with PIC, mixed gently, and centrifuged at 500× *g* at 4 °C (Sorvall C6+ Ultracentrifuge, ThermoFisher Scientific) for 10 min to pellet the guts. Supernatant was removed, and the wash process repeated thrice. Washed guts were split into two halves, to provide a crude extraction control and purified surface protein samples. The control fraction was prepared following the protocol described by Kongsuwan et al. 2010 [28] with the following amendments. *R. microplus* tick guts were suspended and homogenized in 10 mL ice-cold 1× PBS with PIC, followed by sonication (6 pulses, 40 s/pulse) (Branson Ultrasonics Sonifier 250, ThermoFisher Scientific). Tissue homogenates were centrifuged for 20 min at 10,000× *g* at 4 °C to remove remnants, with supernatant collected and centrifuged for a further hour at 100,000× *g* at 4 °C. These final pellets were recovered and designated as the ‘crude extraction control’. 

The remaining half of *R. microplus* tick guts was re-suspended in 10 mL of cell culture medium Dulbecco’s modified eagle medium (DMEM) (Sigma-Aldrich) 2% fetal calf serum (FCS) (Sigma-Aldrich), 0.5 mM ethylenediaminetetraacetic acid (EDTA) (Sigma-Aldrich), 1 mM tris(2-carboxyethyl) phosphine (TCEP) (Sigma-Aldrich), and PIC, and incubated for an hour at 37 °C under slow rotation using a roller at 6 rpm to dislodge epithelial gut cells from the gut membrane. The resulting suspension was filtered through a 250-µm cell strainer (ThermoFisher Scientific), and a second 70-µm cell strainer, with flow-through collected and centrifuged at 500× *g* at 4 °C for 20 min to pellet single epithelial cells. The pelleted cells were re-suspended in 3 mL DMEM. 

### 2.2. Isolation of Single Epithelial Cells and Purification of Surface Proteins 

Single epithelial cells were recovered using a Percoll (Sigma-Aldrich) centrifugation gradient, with the surface proteins biotinylated, and isolated following the protocol described previously [27] with the following amendments. Prior to layering the discontinuous gradient, 6 mL of Percoll was prepared by filtering through an AP15 pre-filter paper, diluted to 3 mL 40% and 3 mL 20% Percoll in MilliQ (v/v) (Merck Millipore, Bayswater, Australia), and cooled at 4 °C for an hour. A peristaltic pump (Easy-Load^®^ Masterflex, Model 7518-10, John Morris Scientific, Murarrie, QLD, Australia) was used to layer the Percoll gradient at a flow rate of <1 mL/min. Three millilitres of 40% Percoll was pumped into a 16-mL ultracentrifuge tube on ice, to constitute the lower layer. The lower layer was allowed to cool and settle on ice for 15 min prior to layering the second 20% Percoll layer over the 40% layer. The final layer, consisting of 3 mL DMEM containing tick gut cells (from Section 2.1 above) was layered over the 20–40% Percoll gradient. Utilizing an ultracentrifuge (Sorvall C6+ Ultracentrifuge, ThermoFisher Scientific), programmed for maximum acceleration and minimum deceleration, the gradient was centrifuged at 600× *g* for 10 min at 4 °C. The interphases between DMEM:20% Percoll, and 20:40% Percoll containing the isolated epithelial cells were collected and stored at 4 °C. 

Isolated epithelial cells were biotinylated using a Biotin (Type A) conjugation kit (ABCAM, Wetherill Park, NSW, Australia), at a molar ratio 1:1 surface protein to conjugate as per the manufacturer’s instructions. Cell lysis was performed by adding 100 µL of 1× PBS, 1% Triton X-100, 10% glycerol, 100 µm oxidized glutathione and PIC to the biotinylated cells, incubated for an hour on ice, with gentle agitation every 10 min. Biotinylated cells were centrifuged at 20,000× *g* at 4 °C for 20 min, with the supernatant containing the cytoplasmic and biotinylated surface proteins collected. Streptavidin magnetic beads (New England Biolabs, Arundel, QLD, Australia) were used to purify the biotinylated surface proteins from the cytoplasmic proteins as per the manufacturer’s instructions. The quantity of proteins extracted was determined via a Bradford Assay (Bio-Rad, Gladesville, NSW, Australia) as described by the manufacturer. 

### 2.3. LC/MS Analysis of Biotinylated Gut Surface and Crude Gut Proteins

Surface proteins, isolated from crude gut or isolated epithelial cells, were identified through liquid chromatography-mass spectrometry (LC/MS) analysis. The biotinylated surface proteins, isolated from the tick gut epithelial (from Section 2.2 above) were electrophoresed on a 4–20% Tris-3-(N-morpholino) proprane sulfonic acid (MOPS) Gel (GenScript, Piscataway, NJ, USA) and visualized using SilverQuest Staining Kit (Invitrogen, Mt Waverley, VIC, Australia) following the manufacturer’s instructions. Using a size 11 scalpel, lanes were divided into fragments and placed into 1.5 mL Eppendorf tubes. Gel fragments were crushed using the flat edge of the scalpel blade, and destained according to the manufacturer’s instructions. 

Destained gel fragments were centrifuged at 15,000× *g* for 1 min, destain solution removed, and gel fragments reduced in 40 µL dithiothreitol (DTT) at 60 °C for 30 min. Following reduction, gel fragments were centrifuged at 15,000× *g* for 1 min, and supernatant removed. Free cysteine residues were alkylated by adding 40 µL iodoacetamide and incubated for 30 min at room temperature in the dark. Gel fragments were centrifuged at 15,000× *g* for 1 min and the iodoacetamide solution was removed, followed by three washes in 100 µL of 50 mM ammonium bicarbonate buffer (ABC). The gel fragments were dehydrated by the addition 100 µL 100% acetonitrile (ACN) (Sigma-Aldrich) and incubated at room temperature with agitation until they became white in appearance, and had shrunk in size by ~50%. The gel fragments were rehydrated in 8 µL trypsin (10 ng/µL in 50mM ABC) (Promega, Alexandria, NSW, Australia) with incubation for 20 min at 4 °C. An additional 30 µL of 50 mM ABC buffer was added to the gel fragments, with the fragments incubated overnight at 37 °C. Peptide extraction was conducted by adding 50 µL 0.1% trifluoroacetic acid (TFA) (Fluka, Gillman, SA, Australia)/50% ACN followed by sonication in a water bath at room temperature for 10 min (Branson Sonifier 250, Fisher Scientific). Gel fragments were centrifuged at 15,000× *g* for 1 min, with supernatant collected and peptide extraction repeated for a total of three extractions. The supernatant from each protein extraction was pooled and lyophilized in a concentrator plus speedvac (Eppendorf, Macquarie Park, NSW, Australia) at 45 °C for 4 h, or until the supernatant was fully evaporated. Dried samples were resuspended in 10 µL of 5% ACN/0.1% TFA, and desalted using C18 ZipTips (0.6 µL resin) as per manufacturer’s instructions (Millipore C18 ZipTip, Sigma-Aldrich) with a final elution in 10 µL 80% ACN/0.1% TFA. 

Samples were separated using reversed-phase chromatography on a Shimadzu Prominence nanoLC system (Shimadzu, Sydney, NSW, Australia). Using a flow rate of 30 µL/min, samples were desalted on an Agilent C18 trap (0.3 × 5 mm, 5 µm) (Agilent, Mulgrave, VIC, Australia) for 3 min, followed by separation on a Vydac Everest C18 (300 A, 5 µm, 150 mm × 150 µm) (BGB Analytik, Alexandria, VK, USA) column at a flow rate of 1 µL/min. A gradient of 10–60% buffer B over 45 min where buffer A = 1% ACN/0.1% Formic Acid (FA) (Sigma-Aldrich) and buffer B = 80% ACN/0.1% FA was used to separate peptides. Eluted peptides were directly analyzed on a TripleTof 5600 instrument (ABSciex, Mulgrave, VIC, Australia) using a Nanospray III interface. Gas and voltage settings were adjusted as required. Time-of-Flight mass spectrometry (MS TOF) scan across *m*/*z* 350–1800 was performed for 0.5 s followed by information dependent acquisition of the top 20 peptides across *m*/*z* 40–1800 (0.05 s per spectra), with resulting data searched using ProteinPilot 5.0.1 (Sciex, Mulgrave, VIC, Australia) to identify proteins. Sequences were identified using a database populated from *R. microplus* sequences, *Ixodidae* Swiss-Prot and Translated European Molecular Biology Laboratory Nucleotide Sequence Database (TrEMBL) sequences from UniProt (October 2017) and the *R. microplus* draft genome sequence [29]. To determine the presence of host proteins, sequences were also identified using a database populated from *Bos* spp. Swiss-Prot and TrEMBL sequences from UniProt (October 2017). Search parameters were set as Special Factors: Gel-based ID, ID Focus: biological modifications, Search Effort: thorough ID. Protein identifications were manually verified and required a protein to have at least one unique peptide with a contribution of >1.3 at 95% confidence. Identified proteins were collated into FASTA format and analyzed with BLAST2GO (BioBam, Valencia, Spain) to retrieve annotations, gene oncology (GO) terms, InterPro IDs, and to remove duplicate identifications. Membrane localization was confirmed through TMHMM Server v2.0 [30] and PredGPI [31]. 

## 3. Results

### Purification of Biotinylated Membrane-Bound Protein and LC-MS/MS Analysis

**Figure 2:**
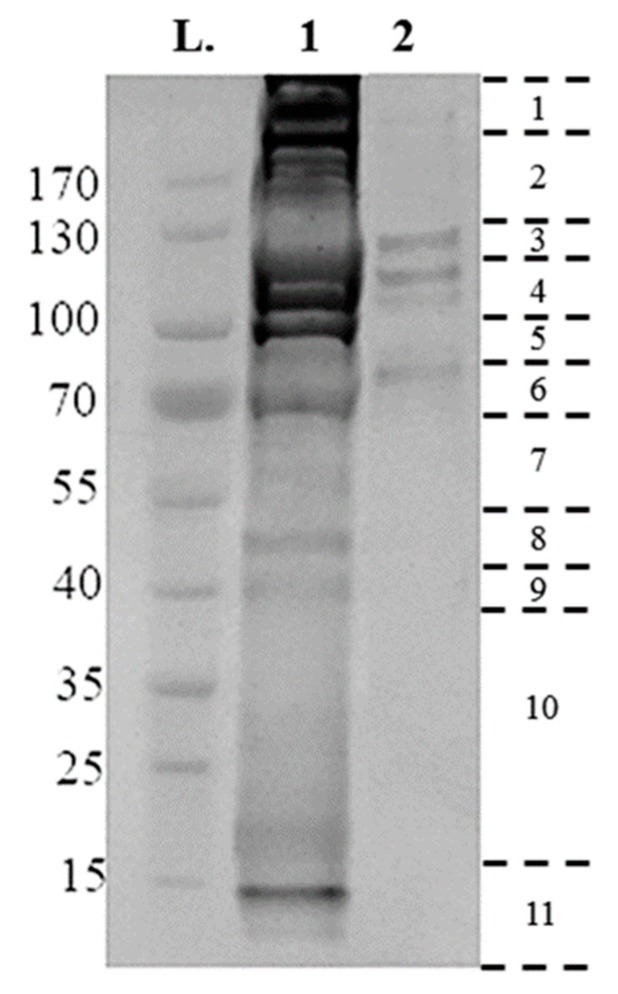
Electrophoretic separations of surface proteins purified from *Rhipicephalus microplus* tick guts, run on a 4–20% tris- 3-(N-morpholino) proprane sulfonic acid (MOPS) gel, at 140 V for 55 min using 10 µg of sample. Gel lanes were divided into eleven fragments, as indicated at the right, with the resulting fragments reduced, alkylated and digested, and analyzed by liquid chromatography-mass spectrometry (LC-MS/MS). (**L**) Page Ruler Prestained Protein Ladder; (**1**) Crude Extraction tick-preparation; (**2**) Membrane-bound protein extraction.

Utilizing the protocol described above, 25 µg membrane-bound and 75 µg crude gut proteins were extracted from 50× semi-engorged *R. microplus* guts per sample preparation. Then, 10 µg of crude gut proteins and membrane-bound proteins were electrophoresed on a 4–20% Tris-MOPS Gel (Figure 2). Band patterns were different among the samples, as the crude gut proteins contain a complex mixture of proteins from both host and tick, with a broad range of molecular sizes from 15 to >170 kDa. 

The membrane-bound proteins consist of a more succinct range of proteins contained within four bands of 80, 110, 120, 130 kDa in size (Figure 2). Proteins were identified by searching databases with the combined spectra of the 11 gel slices from each fraction, with searches performed in two databases: A tick-protein database formed from *Ixodidae* UniProt (October 2017) and the *R. microplus* draft genome [29], and a host-specific *Bos* spp. UniProt (October 2017). Table 1 summarizes the number of proteins identified from each extraction, and their localization as determined by InterPro ID, TMHMM, and PredGPI. Crude extraction methodology identified 692 tick specific proteins, of which 108 were identified as membrane-bound, with 824 *Bos* spp., host proteins. Conversely, the technique as described previously [27] identified 1242 tick-specific proteins, of which 314 were identified as membrane-bound, with 595 *Bos* spp. host proteins. 

**Figure 3:**
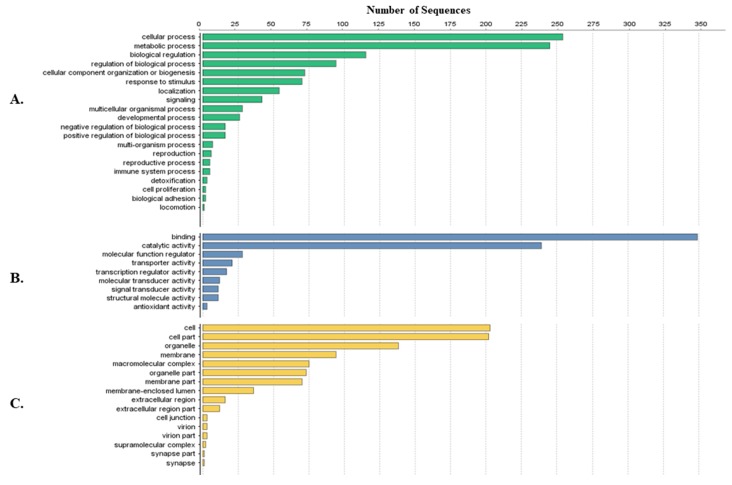
Protein characterization of tick-specific proteins identified after applying the crude extraction methodology as described by Kongsuwan et al. 2010 [28]. Figures were created through BLAST2GO software, displaying the 20 most identified GO terms. (**A**) Biological Process; (**B**) Molecular Function; (**C**) Cellular Component.

**Figure 4:**
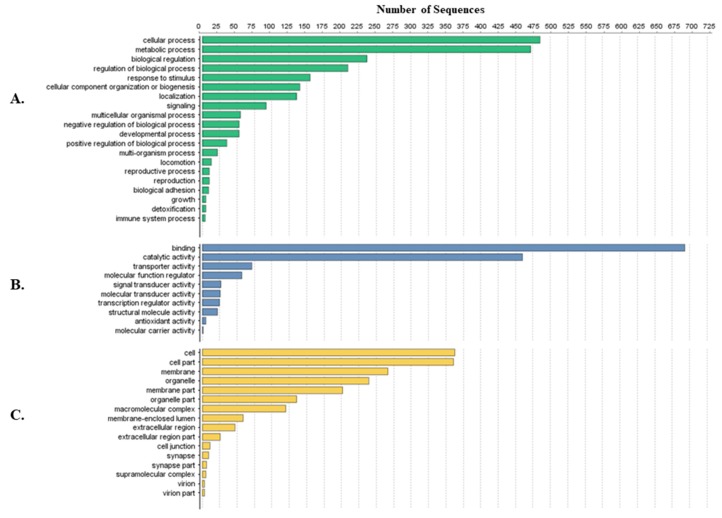
Protein characterization of tick-specific proteins identified after membrane-bound methodology as described by Karbanowicz et al. 2017 [27]. Figures were created through BLAST2GO software, displaying the twenty most identified GO terms. (**A**) Biological Process; (**B**) Molecular Function; (**C**) Cellular Component.

**Figure 5:**
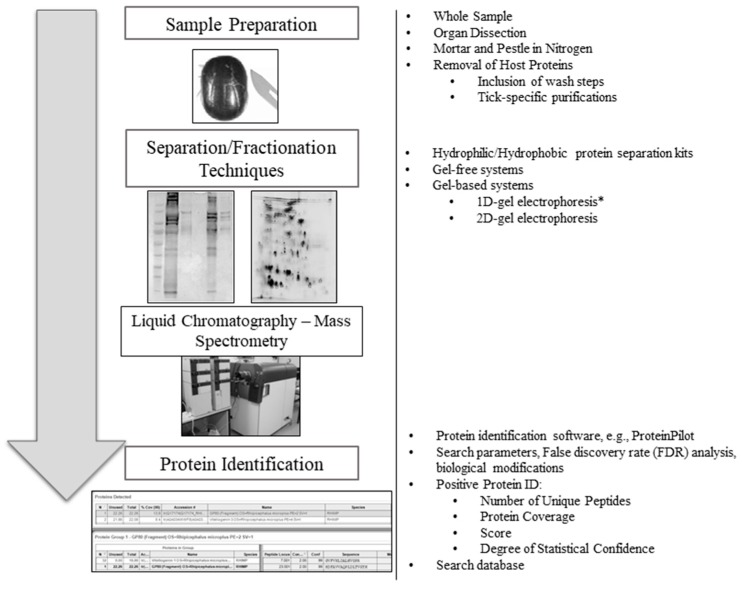
An overview schematic workflow utilized for tick proteomic analyses to date. To the right of each step is an overview of the variety of methods previously used in tick proteomics. Two-dimensional gel image source [32] (*) One-dimensional gels are the preferred method for tick proteomics analysis, as previously described by Madden et al. [32].

Tick-specific proteins were functionally characterized through BLAST2GO software and GO term analysis. Overall, the biological process, molecular function and cellular component terms between both crude (Figure 3) and membrane-bound (Figure 4) samples were similar. Variations in the number of identified sequences were observed between the sample preparations, however no significant incongruity in GO terms was observed. From a total of 692 and 1242 proteins identified, 350 and 682 proteins were characterized from crude and membrane-bound sample preparations respectively. Figure 5 provides a schematic workflow which to date have been applied for tick proteomic investigations including this study which focusses on the use of 1D gel separation techniques. The overall quantity and quality of proteins identified through proteomics is dependent upon sample preparation, separation and fractionation, and ultimately the vigor of the database applied for protein identification.

## 4. Discussion

Proteomics are a tool for the characterization of dynamic interactions that cannot be analyzed by genomic, sialomic, or transcriptomic approaches alone [33]. In contrast to genomic studies, there is no single static proteome in any organism. An organism’s proteome is a dynamic array of proteins in different cells and tissues that display variations in response to conditions such as stress, infectious processes, or specific to ticks, the physiological changes throughout engorgement, and life cycle stages [33,34,35,36]. The use of proteomic approaches for the identification of vaccine candidates provides an important impetus for the continual development of proteomic technologies, and species-specific methodologies. A variety of methods have been used in tick proteomic studies, with few studies conducting a comparison, and their influence on the final protein quantities and qualities identified. Furthermore, the parameters constituting a positive protein identification varies between identification software and reported studies. 

The first stage in any proteomic workflow is sample preparation. Early tick proteomic experiments pulverized whole ticks (often not freshly collected or stored frozen), after freezing in liquid nitrogen, in an attempt to identify and analyze the proteome. These investigations however were limited, as the cuticle contributes to most of the tick’s total mass, and as such these proteomic investigations principally identified cuticle proteins, such as chitin, actin, tubulin and keratin [36,37]. The abundance of these structural proteins hinders the detection of low abundance proteins, resulting in a low variety of tick specific proteins identified. Later experiments dissected freshly collected ticks to retrieve major tick organs, improving the specificity and diversity of tick protein discovery. Through the removal and separate analysis of the cuticle, viscera and salivary glands, a broader and more comprehensive proteome is defined [23,24,25,35]. Although organ-specific preparations were an improvement over whole-tick preparation, the presence of tick, host, and vector-borne symbiont proteins in tick samples remained. This has further driven the investigation of sample preparation methods, to isolate tick-specific proteins from this complex mixture. The crude extraction methodology used for this study was first described by Kongsuwan et al. 2010 [28] and has been used as the principal method for sample preparation for several proteomic investigations of tick midguts [23,24,28]. This methodology employs the use of various wash and centrifugation steps in the attempt to purify tick-proteins from the host proteins contained within the blood meal. Although this method was an improvement on whole-tick and organ-specific preparations, in our study, this method only identified 824 host proteins and 692 tick proteins. The presence of host proteins can lead to the mis-identification of tick specific proteins and decreases the total unique proteins identified by overloading the column with host proteins. In comparison, the purification of biotinylated membrane-bound proteins from isolated epithelial cells as described in this study identified 1242 tick-specific proteins, with only 595 host proteins. The methodology approximately doubled the amount of tick-specific proteins identified at 95% confidence, whilst reducing the background contamination from host proteins contained within the blood meal. 

As the mid-gut is principally investigated to identify membrane-bound vaccine candidates, it is important to note whether either sample preparation method is suitable. Crude extraction methods identified 108 membrane-bound proteins, whereas the novel extraction method described here identified 314, indicating that either method is suitable for the investigation of membrane-bound proteins however, further investigation is required to determine whether the proteins identified are suitable vaccine candidates. As indicated by GO-term functional analysis, the proteins identified between both extractions were similar in function with no significant difference between crude and membrane-bound preparations. From a total of 692 and 1242 proteins identified, 350 and 682 proteins were characterized from crude and membrane-bound sample preparations, respectively, highlighting the continual need to characterize and annotate tick-proteins. The continual development of tick-specific methods is important and our results indicate that our cell surface isolation technique [27] is a suitable replacement to crude extractions for the proteomic investigation of tick mid-guts. 

Proteomic preparations are a multi-step process with sample preparation defining one of the three key stages that require development of tick-specific methods. Following sample preparation is the separation and fractionation of proteins commonly conducted through electrophoresis. Characterization of protein profiles began 25 years ago with the introduction of two-dimensional (2-D) gels capable of separating complex mixtures present in cells [34,38]. Classic proteomics relies upon gel-based electrophoresis to separate proteins on either a one-dimensional (1-D) or 2-D gel for analysis of either all proteins or a subset. These separation techniques are experimentally restricted as gel-based systems are limited to proteins of 10–120 kDa with neutral-acidic isoelectric points, have difficulty displaying hydrophobic proteins, and proteins of low abundance, making the identification and characterization of a comprehensive proteome difficult [39,40,41,42]. Gel-free “shotgun” proteomics techniques have since replaced gel-based separation as the preferred method of sample preparation. In gel-free systems samples are digested in solution and separated through chromatography, without the need for gel-based electrophoresis [42]. Despite gel-free systems limiting the loss of sample and separating the samples without the prejudice of gel-based systems, their implementation in tick-specific proteomics has been slow. 

The decisive step in any proteomic investigation is the identification of proteins through identification software. Software matches the spectra data for the peptides identified to a protein from a protein sequence database. Currently, the lack of available sequence information for ticks constrains the accurate identification of proteins. Recent sequencing of the genome of *Ixodes scapularis* [43], and the release of transcriptomic data from other medically or agriculturally important species such as *Rhipicephalus*, *Dermacentor*, and *Amblyomma* species have increased the number of tick proteins in the UniProt database from 26,066 proteins in January 2014 (taxonomy *Ixodida*) [22,44] to 183,106 proteins in January 2018. 

The significance of this additional data is observed in the investigation of *Ornithodorus erraticus*, and *O. moubata* by Oleaga et al. [24,25]. Here, the same team of researchers conducted identical sample preparation, separation, fractionation, and final protein identification through Mascot Daemon and Mascot services. In the initial study, 555 unique tick proteins were identified from the midguts of *O. erraticus*, whereas the later study identified 1491 unique tick proteins from the midguts of *O. moubata* [23,24]. Despite the investigations being conducted on two different tick species, the sharp rise in protein identification correlates with the sharp increase in available tick-specific sequences. There is a current need for more genomic, and transcriptomic information to populate and generate more robust databases to facilitate the identification of previously unknown proteins, however the gradual increase in this sequence data should prompt researchers to re-investigate earlier tick-proteomic investigations. 

## 5. Conclusions

Proteomics are a powerful tool for the characterization of the dynamic array of proteins in different cells and tissues, however their use in tick-specific investigations is still novel and under development. The constant expansion of tick-specific methods for proteomic investigations is important, with the lack of sequence databases for ticks, and the presence of host proteins presenting as the major limitations. Within this study, a novel method for the purification of surface proteins from the midguts of *R. microplus* was described, to reduce the presence of host proteins in proteomic investigations. Although not repeated in this study, we recommend the use of biological replicates in order to substantiate important discoveries from the tick proteome.

## Figures and Tables

**Table 1:** Summary of unique proteins identified from semi-engorged *Rhipicephalus microplus* crude gut extraction, and purification of membrane-bound proteins from isolated *Rhipicephalus microplus* gut cells. Positive identification of proteins required at least one unique peptide with a contribution of >1.3, at 95% confidence. Membrane localization was confirmed through gene oncology (GO) term, TMHMM, and PredGPI analysis.

	Crude Extraction	Membrane-Bound
Total tick proteins	692	1242
Total membrane-bound	108	314
Total host proteins	824	595
